# Multi-visceral resection of pancreatic VIPoma in a patient with sinistral portal hypertension

**DOI:** 10.1186/1477-7819-6-80

**Published:** 2008-07-28

**Authors:** David L Joyce, Kelvin Hong, Elliot K Fishman, Joshua Wisell, Timothy M Pawlik

**Affiliations:** 1 Departments of Surgery, Johns Hopkins School of Medicine, Baltimore, MD, 22187-6681, USA; 2 Department of Interventional Radiology, Johns Hopkins School of Medicine, Baltimore, MD, 22187-6681, USA; 3 Department of Radiology, Johns Hopkins School of Medicine, Baltimore, MD, 22187-6681, USA; 4 Department of Pathology, Johns Hopkins School of Medicine, Baltimore, MD, 22187-6681, USA

## Abstract

**Background:**

VIPomas are rare neuroendocrine tumors poorly described in the literature. Aggressive resection of patients with advanced VIPoma neuroendocrine tumors has rarely been reported.

**Case presentation:**

A 46 year old women presented with abdominal pain and diarrhea. A three-dimensional (3-D) pancreas protocol computed tomography scan revealed an 18 × 12 cm pancreatic VIPoma abutting the liver, stomach, spleen, left adrenal, colon that also invaded the distal duodenum – proximal jejunum at the ligament of Treitz in association with sinistral portal hypertension. Following preoperative proximal splenic artery embolization, the patient with underwent successful en bloc resection of the locally advanced VIPoma in conjunction with a diaphragmatic resection, total gastrectomy, splenectomy, left adrenalectomy, as well as small and large bowel resection. The estimated blood loss was 500 ml. All margins were negative (R0 resection). The patient is alive and disease-free.

**Conclusion:**

This case illustrates the role of aggressive resection of pancreatic neuroendocrine tumors and highlights several key technical points that allowed for successful resection.

## Background

VIPomas are rare neuroendocrine tumors with an annual incidence of about 1 per 10,000,000 individuals.[1] The majority of VIPomas in adults (> 90%) are primary tumors of the pancreas.[2] As with other neuroendocrine tumors of the pancreas, on occasion these lesions can be exceptionally large with invasion of adjacent visceral and vascular structures. As such, accurate preoperative imaging is critical. In particular, assessment of the relationship between the tumor and adjacent vascular structures, such as the portal and superior mesenteric vein (SMV) as well as the celiac and superior mesenteric artery (SMA), is critical in determining preoperative resectability. On occasion, invasion of the tumor into the adjacent splenic-portal venous system can lead to sinistral, or left-sided, portal hypertension.

Surgical resection of pancreatic VIPoma provides the only chance at long-term cure, as systemic chemotherapeutic agents are associated with poor response rates.[3] Nevertheless, aggressive resection in patients with advanced VIPoma neuroendocrine tumors has rarely been reported. While part of the reason for this undoubtedly is due to the rarity of VIPomas, another factor may be related to the reluctance to perform aggressive resection due to possible increased morbidity and mortality.[4] With careful attention to pre- and intra-operative details, aggressive resection of VIPomas can be accomplished safely, thereby providing the patient with an opportunity for extended long-term survival. We herein report a case of multi-visceral resection of pancreatic VIPoma in a patient with sinistral portal hypertension. Furthermore, we provide a brief review of the role of aggressive resection of pancreatic neuroendocrine tumors and highlight several key technical points that allowed for successful resection.

## Case presentation

A 46-year-old obese woman presented to an outside hospital in August of 2005 with significant abdominal pain and diarrhea. Computed tomography (CT) revealed a 17 × 13 cm mass in the left upper quadrant that appeared to arise from the body and tail of the pancreas. The patient was taken to the operating room at an outside institution, but the mass was deemed unresectable due to reported involvement of the SMA, stomach, and colon. Wedge biopsy of the mass was consistent with pancreatic VIPoma. Over the next 2 years, the patient was treated with long-acting somatostatin with some improvement in her symptoms. The patient, however, developed repeat episodes of upper and lower gastrointestinal bleeding with associated anemia and ongoing transfusion requirements. Repeat CT scan revealed thrombosis of the splenic vein with numerous large splenic and gastric varices consistent with sinistral portal hypertension. In the summer of 2007, the patient underwent a failed transjugular intrahepatic portosystemic shunt (TIPS) procedure at an outside institution. The patient was therefore referred to the Johns Hopkins Department of Interventional Radiology for variceal embolization.

**Figure 1:**
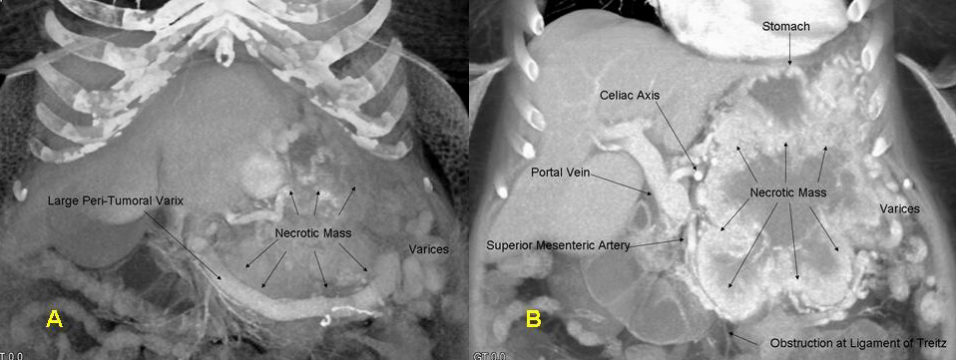
**(A) 3-D CT coronal reconstruction showing the pancreatic VIPoma, a large peri-tumoral varix, and gastric varices**. **(B) **3-D CT coronal reconstruction depicting relation of pancreatic VIPoma to adjacent vascular structures and stomach. Note presence of varices as well as invasion of tumor into the fourth portion of the duodenum.

**Figure 2:**
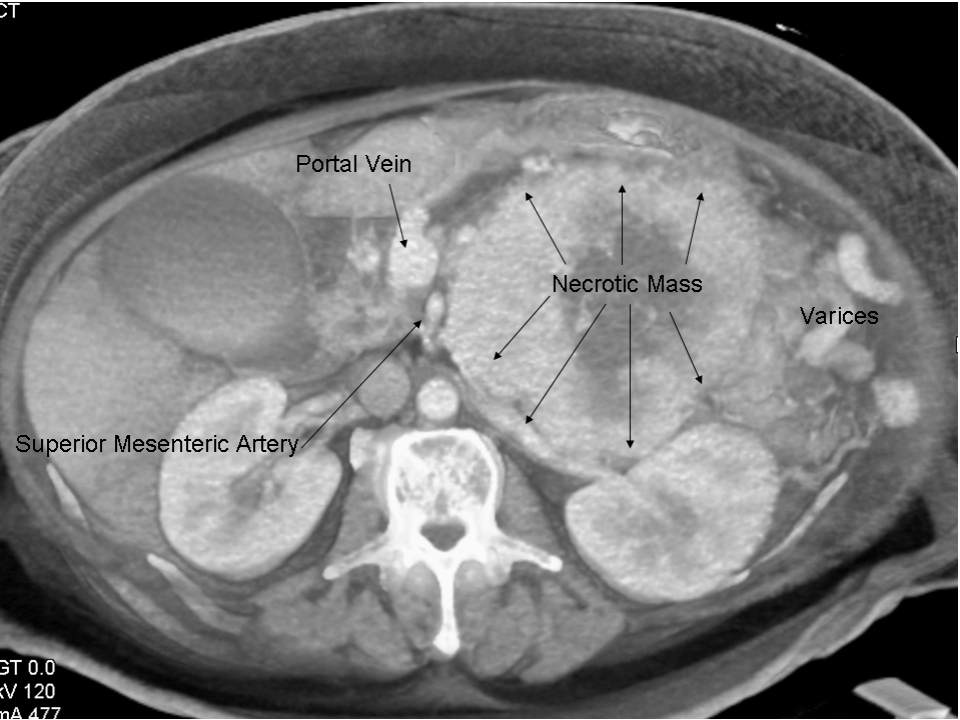
**Cross-sectional CT depiction of large necrotic pancreatic VIPoma and its relation to the portal vein and superior mesenteric artery**.

The patient's case was reviewed at the Johns Hopkins multi-disciplinary pancreas tumor board. A repeat three-dimensional (3-D) pancreas protocol CT scan revealed an 18 × 12 cm mass abutting the liver, stomach, spleen, left adrenal, colon and invading the distal duodenum – proximal jejunum at the ligament of Treitz. The splenic vein was occluded. Large collateral vessels surrounded the mass and were associated with extensive gastric collaterals (Figure 1). The mass displaced the SMA and SMV, but these vessels were patent and uninvolved (Figure 2). As such, there were no obvious contraindications to resection and surgery was recommended.

**Figure 3:**
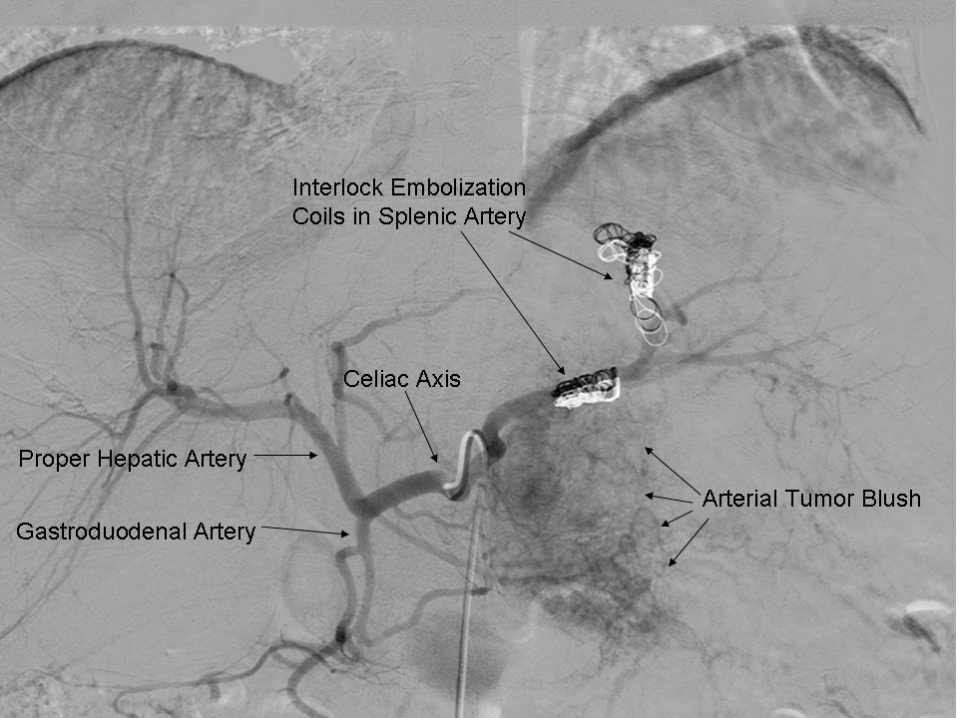
**Celiac axis arteriogram depicting normal arterial anatomy and presence of interlock embolization coils used to embolize the proximal splenic artery preoperatively**.

**Figure 4:**
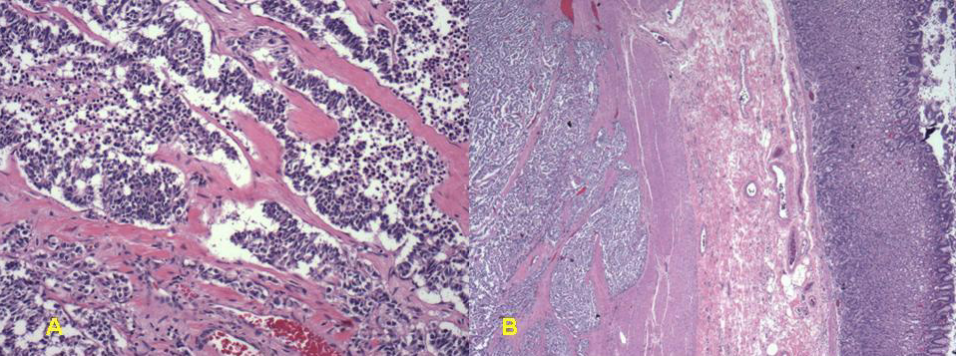
**(A) Typical of pancreatic neuroendocrine tumors, this lesion contains interconnecting nests and trabeculae of uniform cuboidal cells with granular cytoplasm and central round nuclei within a hyalinized, well-vascularized stroma (Original magnification ×100)**. **(B) **The tumor deeply invades the muscularis propria of the stomach (Original magnification ×20).

Given the size of the mass and the associated extensive varices, the patient underwent preoperative proximal splenic artery embolization (Figure 3). Twenty-four hours following this, the patient was taken to surgery where she was found to have a very large mass arising from the body and tail of the pancreas that invaded the left diaphragm, stomach, left adrenal, fourth portion of the duodenum – first portion of the jejunum, transverse colon, and spleen. In order to better expose the SMV at the inferior border of the pancreatic neck, the right colon and root of the small bowel mesentery were mobilized in the fashion of Cattell and Braasch. The SMA medial to the SMV was exposed as it coursed into the small bowel mesentery. The tumor was noted to closely abut and displace both the SMV and SMA, but the vessels were not encased. After developing the retro-pancreatic plane over the SMV – portal vein, the pancreatic neck was transected. The mass was subsequently resected en bloc with a portion of the left diaphragm, entire stomach, spleen, left adrenalectomy, fourth portion of the duodenum – proximal jejunum and transverse colon. Gastrointestinal continuity was restored using a Roux-en-Y method with a hand sewn end-to-side esophago-jejunostomy, a duodeno-jejuneal anastomsis (50 cm distally), and a stapled colo-colonic anastomosis. The pancreatic remnant was closed with pledgeted sutures. Estimated blood loss was 500 ml. Final pathology confirmed a VIPoma originating from the pancreatic body with invasion of the stomach, spleen, small bowel, and colon (Figure 4). All margins were uninvolved by tumor. The patient is alive and disease-free.

The patient tolerated the procedure well. On post-operative day four, a swallow study demonstrated a normal post-surgical esophago-jejunal anastomosis with no evidence of leak. The patient was discharged home on post-operative day ten tolerating a post-gastrectomy diet. She received no adjuvant therapy and is currently alive and disease-free at 6 months of follow-up.

## Discussion

VIPomas are rare tumors that have been infrequently reported in the literature.[5] These pancreatic tumors secrete excessive amounts of VIP (Vasoactive Intestinal Peptide), a structural homologue of secretin. Elevated serum VIP levels cause increased intestinal secretion of Na^+^, K^+^, HCO_3_^-^, and Cl^-^, as well as bone resorption, vasodilation, and inhibition of gastric acid section. These effects lead to a well-defined clinical syndrome, characterized by watery diarrhea, hypokalemia, and hypochlorhydria. Despite this, the VIPoma syndrome can be difficult to diagnosis and these tumors can elude prompt diagnosis.[5] As such, similar to other neuroendocrine tumors, VIPomas can be quite large at the time of presentation and involve adjacent structures. As in the current case, locoregional extension can include invasion into visceral structures. However, with an aggressive surgical approach that allows for complete tumor extirpation, extended, meaningful survival can be achieved for VIPoma patients.[5]

Norton *et al*.,[4] have reported that aggressive surgery can be done with acceptable morbidity and low mortality rates for patients with advanced neuroendocrine tumors. In a series of 20 patients with advanced tumors, Norton *et al*.,[4] reported a post-operative complication rate of 30% and no operative deaths. In that study, surgery variably included pancreatectomy, splenectomy, superior vein reconstruction, and liver resection. In the current case, the patient underwent an extensive procedure that included pancreatectomy, splenectomy, total gastrectomy, left adrenalectomy, diaphragmatic resection, as well as small and large bowel resection. An R0 resection (microscopically negative margins) was achieved and the patient did well post-operatively. Patients with locally advanced neuroendocrine tumors that can be technically resected with an R0 margin should therefore be offered surgical resection even when a multi-visceral resection is necessary. In high-volume institutions, these procedures can be accomplished with acceptable morbidity and near-zero mortality.[4,6,7]

Accurate CT imaging is critical in assessing locoregional resectability.[8,9] Recently, 3-D CT scan has been reported to enhance the assessment of the tumor-vascular interface,[10] as the 3-D format allows for better viewing of oblique orientations.[11] Accurate information concerning the relation of the tumor with the SMA is particularly critical as major arterial encasement may preclude an R0 resection. It is important to note, however, that intraoperative assessment of the tumor-SMA relationship can be very limited – especially in patients with large tumors.[12] This is evidenced in the current case in which the initial surgeon deemed the lesion to be unresectable based on an intraoperative assessment that the SMA was encased. High-quality cross-sectional imaging clearly demonstrated, however, that the SMA was indeed not involved (Figure 3). This case highlights how intraoperative assessment of the tumor-SMA interface may be both limited and misleading. Rather, thin-section contrast-enhanced CT should be utilized as the modality of choice in assessing the relationship of the primary tumor to major vascular structures such as the SMV, PV, SMA, and celiac axis. Such determinations have important clinical implications in deciding which patients are candidates for aggressive resection of advanced pancreatic tumors.

For tumors such as the one presented here, the surgeon should still evaluate the SMV and SMA early in the course of surgery. Full exposure of the SMV is mandatory and requires mobilization of the colon and root of the small bowel mesentery to expose the SMV where it lies anterior to the third portion of the duodenum. This mobilization should be carried to the left by incising the omental attachment to the mesocolon. After performing a wide Kocher maneuver, the SMA should similarly be identified at the junction of the third and fourth portions of the duodenum as it courses distally. The connective tissue attachments between the portal vein/SMV and SMA can then be divided, thereby isolating the vessels. This "medial" approach allows for early dissection and evaluation of the critical vascular structures. Once the relation of the tumor to these structures has been established, more lateral dissection along the spleen and tail of the pancreas can be accomplishing with little difficulty. This method of dissecting the SMA and SMV first allows the surgeon to avoid committing to an extensive resection prior to determining whether or not an R0 resection is feasible.[13]

Sinistral, or left-sided, portal hypertension rarely causes gastrointestinal hemorrhage. Although there are many causes of sinistral hypertension, it is usually due to pancreatic pathology that compresses/invades the left portal – splenic venous system.[14,15] Splenic vein occlusion results in back pressure which is transmitted to the short gastric and gastroepiploic veins with subsequent formation of varices. Our patient had extensive gastric and peri-tumoral varices that were associated with ongoing bleeding and transfusion requirements. Management of sinistral hypertension traditionally involves surgical removal of the primary tumor if possible. In the current case, although resection was deemed to be feasible, the risk of intra-operative massive hemorrhage was felt to be considerable given the extent of the varices, as well as the size and location of the primary pancreatic mass. Preoperative proximal splenic artery embolization has previously been shown to be a safe and efficacious portal decompression technique.[16,17] Umeda *et al*., [17] have shown that proximal splenic artery embolization shortened operative time, reduced blood loss, and led to less need for transfusion in living donor liver transplantation recipients. In a separate study, Adams and colleagues[16] assessed the benefit of preoperative control of splenic arterial inflow on intraoperative blood loss in a cohort of patients with splenic venous occlusion and sinistral hypertension secondary to chronic pancreatitis. In this study, the mean reduction in blood loss associated with embolization was 1560 ml. The employment of preoperative proximal splenic artery embolization in the present case undoubtedly contributed to our relatively modest blood loss (~500 ml). In complex cases characterized by large tumors, splenic vein occlusion, and significant left-side portal hypertension with associated varices, preoperative embolization of the proximal splenic artery should be considered to allow for portal decompression as a means to reduce intraoperative blood loss. Preoperative splenic artery embolization should be used selectively, however, as it may have associated risks.[18]

## Conclusion

The current case is a unique example of a rare pancreatic tumor (VIPoma) that highlights several important peri- and intra-operative concepts. Aggressive resection of VIPomas is warranted and may provide the only chance at long-term survival. When done at large volume, experienced centers even complex multi-visceral resections can be done with low morbidity and near zero morality. In the subset of patients with associated severe sinistral hypertension, proximal splenic artery embolization should be considered as a preoperative means to decrease blood loss and improve outcome. Only by utilizing a multi-modality approach that incorporates state-of-art cross-sectional imaging, interventional radiology, and surgery can these complex patients be managed successfully.

## Competing interests

The authors declare that they have no competing interests.

## Authors' contributions

TP collection of data, analysis of data, draft of manuscript, critical revisions of draft, final review of manuscript, DJ collection of data, analysis of data, draft of manuscript, critical revisions of draft, final review of manuscript, KH collection of data (interventional radiology), analysis of data, critical revisions of draft, final review of manuscript, EF collection of data (radiology images), analysis of data, critical revisions of draft, final review of manuscript, JW collection of data (pathology images), analysis of data, critical revisions of draft, final review of manuscript. All authors read and approved the final manuscript.
